# The Effect of Mechanical Pretreatment on the Electrochemical Characteristics of PEO Coatings Prepared on Magnesium Alloy AZ80

**DOI:** 10.3390/ma16165650

**Published:** 2023-08-16

**Authors:** Ján Sovík, Daniel Kajánek, Filip Pastorek, Milan Štrbák, Zuzana Florková, Michal Jambor, Branislav Hadzima

**Affiliations:** 1 Department of Materials Engineering, Faculty of Mechanical Engineering, University of Žilina, Univerzitná 8215/1, 010 26 Žilina, Slovakia; milan.strbak@fstroj.uniza.sk; 2 Research Centre, University of Žilina, Univerzitná 8215/1, 010 26 Žilina, Slovakia; daniel.kajanek@uniza.sk (D.K.); filip.pastorek@uniza.sk (F.P.); zuzana.florkova@uniza.sk (Z.F.); branislav.hadzima@uniza.sk (B.H.); 3 Institute of Physics of Materials, Czech Academy of Sciences, Žižkova 513/22, 61600 Brno, Czech Republic; jambor@ipm.cz

**Keywords:** corrosion, magnesium alloys, plasma electrolytic oxidation, electrochemical impedance spectroscopy

## Abstract

The main objective of this article is to provide new information on the effects of mechanical pretreatment of AZ80 magnesium alloy ground with SiC emery papers of different grain sizes on the plasma electrolytic oxidation (PEO) process and corrosion properties of AZ80 in 0.1 M NaCl solution. Then, the roughness of the coated samples was measured by confocal microscopy. The corrosion properties of the ground and coated surfaces were determined by potentiodynamic polarization (PDP) within 1 h of exposure, and electrochemical impedance spectroscopy (EIS) was performed during 168 h of exposure at laboratory temperature. Consequently, the obtained results of the PDP measurements were evaluated by the Tafel analysis and the EIS evaluation was performed by the equivalent circuit analysis through Nyquist diagrams. The morphology and structure of PEO coatings were observed by scanning electron microscopy (SEM) through the secondary imaging technology, and the presence of certain elements in PEO coatings was analyzed by EDS analysis.

## 1. Introduction

Magnesium alloys have many exceptional properties, such as excellent recyclability, high specific strength, and light weight. Thanks to these facts, they are widely used in a variety of applications and the use of magnesium alloys is expanding in the automotive aerospace, electrical, and biomedical industries [1]. However, all of these advantages are significantly limited by the low corrosion resistance of magnesium and its alloys [2]. This drawback leads to numerous limitations in various fields of industry. Nevertheless, magnesium alloys can be used as the sacrificial anodes. The main influence of the poor corrosion resistance is caused by the synergistic effect of the standard negative potential of Mg −2.36 V with respect to the standard hydrogen electrode (SHE), the mixture of the natural properties of Mg, the presence of impurities (Fe, Cu, and Ni), and the quasi-protective passive surface film consisting of MgO or Mg(OH)_2_, which provides protection against alkaline pH environments [3,4]. To improve this poor corrosion resistance, many coating techniques were applied, including chemical conversion coatings, anodizing, electroplating, electro-less plating, organic coatings, and fluoride-based coatings [5,6].

It seems that surface treatment is an effective means to improve the corrosion properties of the base material. The great significance of biodegradable alloys and powders is given to plasma spraying and also electrodeposition. These techniques allow for the formation of protective coatings on the surface [7,8]. Coatings can ensure the protection of the material surface by providing a protective boundary between the metal and the aggressive environment. Therefore, it is important to develop coatings for metals that are affordable and environmentally friendly, they should be uniform and also adhere [9]. A promising and suitable method to solve the problem for magnesium alloys is plasma electrolytic oxidation (PEO), also known as microarc oxidation and microarc discharge oxidation. Compared to the traditional anodizing process, the PEO process is not expensive and the technique itself is environmentally friendly. However, the process takes place under high voltage where plasma discharges occur. The discharges result in partial fusion of an oxide layer and the formation of an adherent oxide layer with improved corrosion and wear resistance as well as suitable electrical properties and high thermal stability. During the formation of PEO coating, the growth of the coating occurs both inward and outward away from the metal surface. The layer itself adheres extremely well to substrate [10,11,12]. This technique is used on light metals such as Al, Ti, and Mg. The formation of the PEO coating itself is influenced by a whole range of aspects, such as the chemical composition and the concentration of the chemicals used in the electrolyte. In addition, the values of the applied voltage and current in the process are of great importance, and temperature is also significant. Another important factor is the chemical composition of the material used in the PEO process [12]. The addition of alloying elements such as aluminum, manganese, zinc, zirconium, and calcium has a positive effect on corrosion and mechanical properties [12]. According to Khaselev et al. [13], the addition of aluminum and zinc reduces the thickness of PEO coating and bigger discharge channels with rough surfaces are formed. On the other hand, alloying with rare earth metals (RE) can improve the corrosion resistance of Mg alloys. Tekin et al. [14] found that the coating with the presence of Mg_14_Nd_2_Y_1_ β-phase has better corrosion resistance than the coating on AZ31B alloy.

The surface morphology of the Mg alloy plays a major role in the formation of the PEO coating. It was found that the surface roughness has a great importance in the formation and properties of PEO coating. Li et al. confirmed the fact that the electrochemical properties of PEO coating gradually deteriorate with increasing surface roughness, which was also studied by Yoo et al. [15,16].

Due to the insufficient information on the effect of mechanical pretreatment on the corrosion behavior of PEO coatings in the case of magnesium alloys, we investigated the above design principles, using different emery papers with different surface granularity as the pretreatment method. The effect of surface granularity on the corrosion behavior of PEO coatings can have a significant impact on the PEO process itself and provide beneficial improvements for various industrial and scientific sectors. Thanks to these advantages, the application of magnesium alloys can be extended to the whole transportation field, which is characterized by harsh performance conditions such as aggressive environments. The main objective of this research is to gain new knowledge about the influence of the mechanical pretreatment of the surface on the corrosion behavior of PEO coatings, in order to support the formation of the coating itself and to provide better corrosion resistance of the coated material exposed to the harsh conditions of a chloride-containing solution.

## 2. Materials and Methods

### 2.1. Preparation of Samples for Metallographic Analysis

Magnesium alloy AZ80 in the as-cast state was used for the experimental procedures. The chemical composition was determined using the apparatus ARL QUANTX EDXRF and is given in Table 1. Then, the metallographic analysis was carried out. This procedure consisted of grinding with SiC with water-cooled SiC emery papers up to p4000 and subsequent polishing with polishing cloths and simultaneous application of diamond paste intended for grain sizes of 3 μm and 1 μm. In the following step, the prepared samples were etched with acetic/picric acid (2.5 mL acetic acid + 2.1 g picric acid + 5 mL distilled water + 35 mL ethanol) for 3 s, then rinsed with distilled water, degreased with ethanol, and dried with an air stream. The microstructure itself was observed using an optical microscope ZEISS AXIO Imager.A1m, and images were captured using an AxioCam MRc5 digital camera. The final images were created using Axion Vision Rel 4.5 software.

### 2.2. Plasma Electrolytic Oxidation

The PEO process itself was performed after a certain number of surface pretreatments. First, grinding was performed with SiC papers up to p180, p500, p1200, and p4000, respectively, cooled with water.

**Figure 1:**
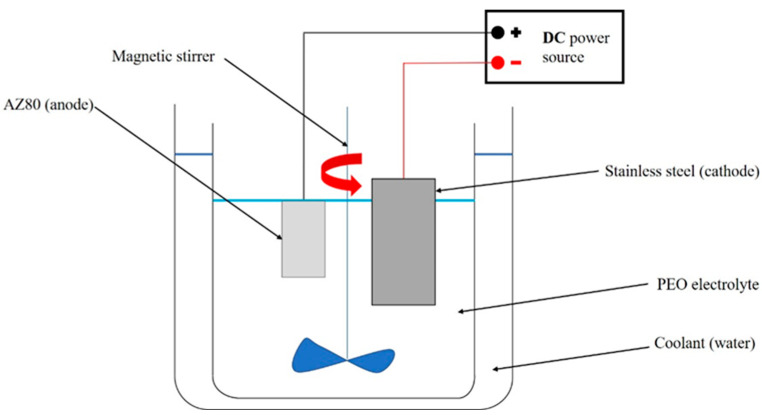
The scheme of the equipment used for the PEO process.

In the second step, PEO treatment was performed. The whole process was carried out in a phosphate-based electrolyte consisting of 12 g Na_3_PO_4_.12H2O and 1 g KOH in deionized water with a pH of 12.5, and the current density was set at 0.05 A/cm^2^. A two-electrode system consisting of AZ80 magnesium alloy connected as an anode and a stainless-steel plate connected as a cathode to a Keysight N8762A (Keysight Technologies Inc., Santa Rosa, CA, USA) DC power source was used in PEO fabrication. However, the constant distance between the electrodes during the application of the coating was set to 10 cm and the magnetic stirrer was inserted into the electrolyte to improve the distribution of reactants relative to the sample surface [17]. Thanks to the container of cold water, containing the PEO electrolyte, the temperature in the electrolyte did not exceed 50 °C. The dimensions of the magnesium samples were 15 mm × 25 mm × 10 mm. The exposure time in the PEO electrolyte was set at 14 min. The schematic of the apparatus used for the PEO process is shown in Figure 1.

### 2.3. Characterization of PEO Surface

The influence of the parameters used during the PEO process on the morphological characteristics of the PEO coatings produced was analysed by scanning electron microscopy (SEM) (TESCAN LYRA3, Brno, Czech Republic), operated at 10 kV. Prior to observation, specimens were coated by a carbon film approximately 10 nm thick. Cross-sections were imaged using backscattered electrons (BSE), and secondary electrons (SE) were used to characterize surface morphology. The chemical composition of the PEO coatings was investigated using the EDS analysis. The average value of the thickness of the PEO coatings themselves was analysed using cross-sectional images. This determination of the average thickness value was performed five times in a row. In addition, these images were also used to evaluate the number and size of pores.

Surface roughness was measured on the coated surface using a Zeiss AXIO Observer Z1 microscope with a confocal head LSM 700 in Zen 2.0 software. The average values were then measured from the measurements, which were performed 10 times in succession, and the following standard roughness parameters are as follows: the arithmetic mean deviation R_a_, the total height of the roughness profile R_z_, and the skewness R_sk_.

### 2.4. Corrosion Testing

**Figure 2:**
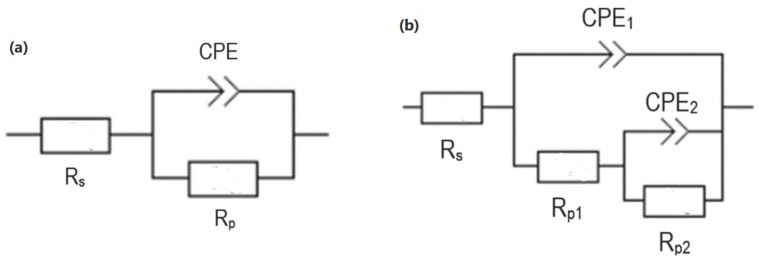
Equivalent circuits for the analysis of Nyquist diagrams with (**a**) one capacitive loop and (**b**) two capacitive loops.

Analysis of the corrosion resistance of AZ80 was performed using electrochemical impedance spectroscopy (EIS). The samples were placed in direct contact with a 0.1 M NaCl solution and the measured area was 1 cm^2^. The test itself took place on a laboratory potentiostat Biologic SP-300 with a corrosion cell attached. For this purpose, the exposure time of the samples was set from 1 h to 168 h at a laboratory temperature of 22 ± 2 °C. The frequency range was set from 100 kHz to 10 mHz, with the frequency change fixed at 10 times per decade. In addition, the amplitude value of the applied sinusoidal potential was set to 15 mV, and the average value of the potential was identical to the open-circuit potential (OCP) value during the EIS measurement itself. A three electrode system was used as the exposure place for EIS tests, which consisted of Mg alloy as the working electrode (WE), a platinum electrode as the counter electrode (CE), and a saturated calomel electrode (SCE) as the reference electrode. To avoid possible errors in the interpretation, all experiments were repeated three times. Corrosion parameters were determined using Nyquist diagrams, which were quantitatively evaluated using the equivalent circuit technique with EC-Lab V11.10 software [5,17,18]. The schematics of circuits used are shown in Figure 2. Figure 2a shows a simple Randles circuit that was used to analyze plots with one capacitance loop, while Figure 2b shows a more complex circuit that was used to analyze plots with two capacitance loops. The significance of two loops is to describe the occurence of areas with different electrochemical behavior. The element Rs in the circuits represents the resistance of the solution, and the element CPE expresses a constant phase element and is defined as [19]:

(1) $$CPE\approx C=[C{\left(j\omega \right)}^{n}{]}^{-1},$$

where the symbol n is described as follows: When n is equal to 1, the CPE is a pure capacitance, when it is equal to 0, it is a pure resistance at this moment used instead of the capacitor (C). Moreover, the next meaning of these elements is to describe heterogeneity of the surface (electrochemically active surface) or the inhomogeneous composition, and the value of CPE plays an important role in this case [20,21]. The element R_p_ is called polarization resistance and it is the most important element due to the fact that it is used in circuit to analyze its corrosion resistance. The value of corrosion resistance is influenced by the value of R_p_. If the Nyquist plot consists of two capacitive loops, the final corrosion resistance of the surface is the sum of the partial resistances R_1_ and R_2_. In the case of the simple circuit with one capacitive loop, the element R_1_ expresses the interface between metal and electrolyte and corresponds to the pore resistance. In the more complex circuit with two capacitive loops, it describes the resistance of the pores, which are typical for the further layer of the coating. On the other hand, the element R_2_ represents the resistance for charge transfer in the inner layer of the PEO coating [4,5,22,23].

The second method used for corrosion testing was potentiodynamic polarization (PDP). This method was carried out in the 0.1 M NaCl solution at the laboratory temperature of 22 ± 2 °C, with the potential range set to the value from −200 mV to +500 mV versus OCP, the rate of potential change was set on the value 1 mV.s^−1^ and the exposure time was 1 h. The Tafel analysis of the potentiodynamic curves was performed using the EC-Lab V11.10 software and the values of following electrochemical parameters corrosion potential (E_corr_), corrosion current density (icorr), coefficients (β_a_ and β_c_), and corrosion rate (r_corr_) were achieved [24].

## 3. Results and Discussion

### 3.1. Metallographic Analysis and SEM with EDS Analysis of Magnesium Alloy AZ80

**Figure 3:**
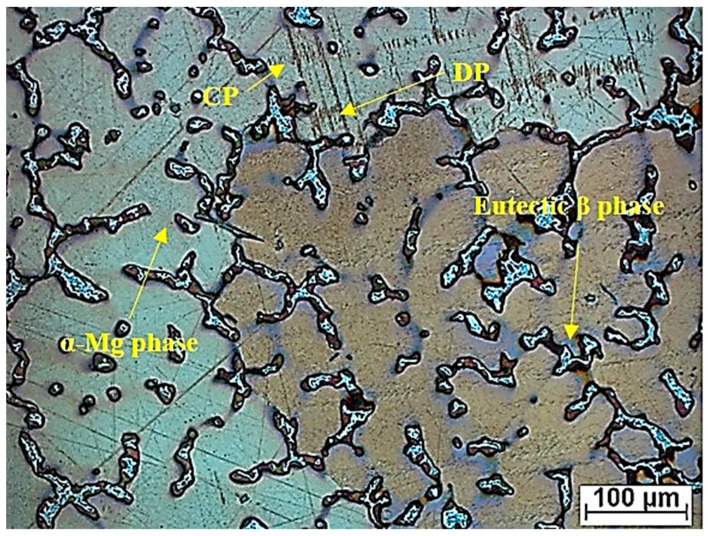
Microstructure of AZ80 magnesium alloy.

Figure 3 shows the microstructure of the experimental material. The microstucture of the as-cast AZ80 magnesium alloy is characterized by a dendritic structure consisting predominantly of a solid solution α-Mg matrix, a small amount of Al_8_Mn_5_ phases, and interdendritic eutectic β—Mg_17_Al_12_ phases, which are distributed along the dendrites. Typical of the Al8Mn5 particles is the fact that they are mainly arranged within the grains. The morphology of β-precipitates is characterized by discontinuous (DP) and continuous precipitates (CP). In addition, the microstructure of AZ80 consists of a certain amount of lamellar and short lath-shaped precipitates, which appear near the eutectic phases after the precipitation process [25].

### 3.2. Mechanism of PEO Formation

The whole PEO process is characterized by the formation of PEO coating, which increases the corrosion protection of metals. For this reason, this type of coating is essential and beneficial for metallic materials, especially light metals. Unfortunately, the pores, which are a typical feature of the structure of PEO coatings, are a major drawback that negatively affects the protective function of the PEO coating [26].

**Figure 4:**
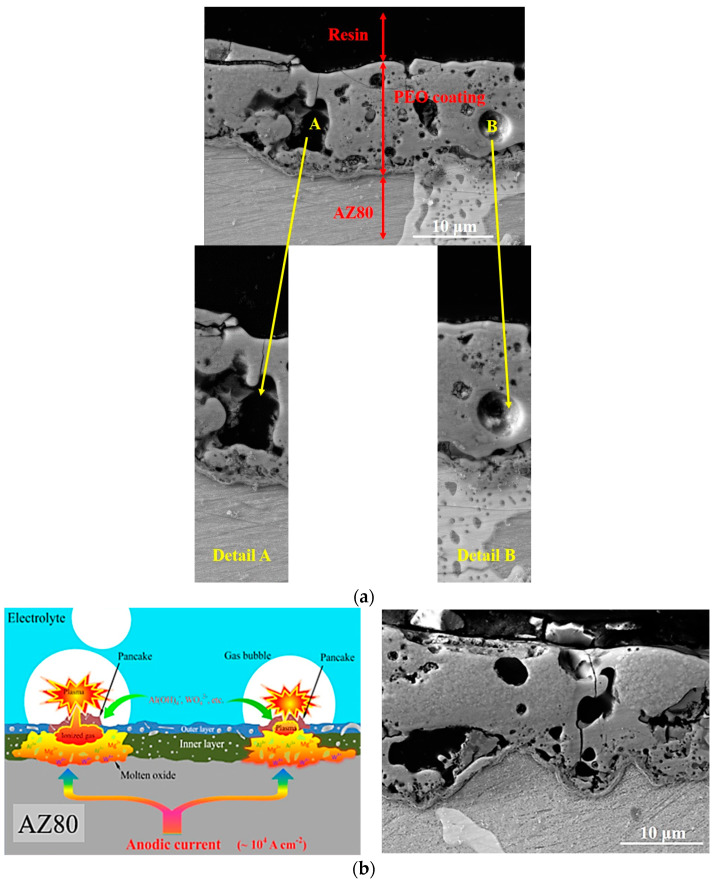
SEM images of PEO coatings—(**a**) PEO coatings with structural drawbacks (pores); (**b**) mechanism of the PEO formation.

In Figure 4b (right one) the mechanism of the PEO formation is shown. The sample is exposed during the process in a PEO bath under the specified conditions (concentration and chemical composition of the electrolyte, exposure time, and values of applied current and voltage). Thanks to these conditions, it is possible to adjust them and achieve the appropriate parameters (thickness and porosity), which are crucial for PEO coating. Due to this, it is inevitable to choose suitable conditions to avoid structural defects or to know how to deal with them or their excessive number and size. Figure 4a,b shows the cross-sections of various SEM images of PEO coatings, where the defects (pores) of the coatings can be seen. The size of these pores is a function of the discharge density as well as the process time. In the case of magnesium alloys is the size between 0.5 and 50 μm [12]. Plasma discharges in PEO processing are limited by the stress behaviour. This fact was demonstrated by Hussein, who found that the PEO process can be divided into four discharge stages. The occurrence of discharges is mainly affected with increasing processing time of PEO [27,28]. Considering these discharges, their intensity, and size, they also have a great importance for the pore formation in the case of the experimental material AZ80 (Figure 4a—detail A and B). While in Figure 4a Detail A, a larger pore with longitudinal shape and dimensions of 12 μm width and 8 μm length can be seen. On the other hand, Figure 4a Detail B shows a spherical pore with a diameter of 4 μm in diameter. Therefore, it should be noted that the formation of the resulting pores in the PEO structure is different in terms of the size and amount of these defects. Figure 4b on the left shows the typical mechanism of PEO formation. The process itself is accompanied by various process characteristics, such as developed voltage breakdown, local melting and oxidation of the substrate, and quenching and recrystallization processes, which have a significant impact on the resulting properties of the coating [29]. In the Figure 4b, right, the cross-section of Mg alloy AZ80 after PEO treatment is shown. The result of this mechanism is the porous structure of the PEO coating.

### 3.3. Voltage Behaviour

**Figure 5:**
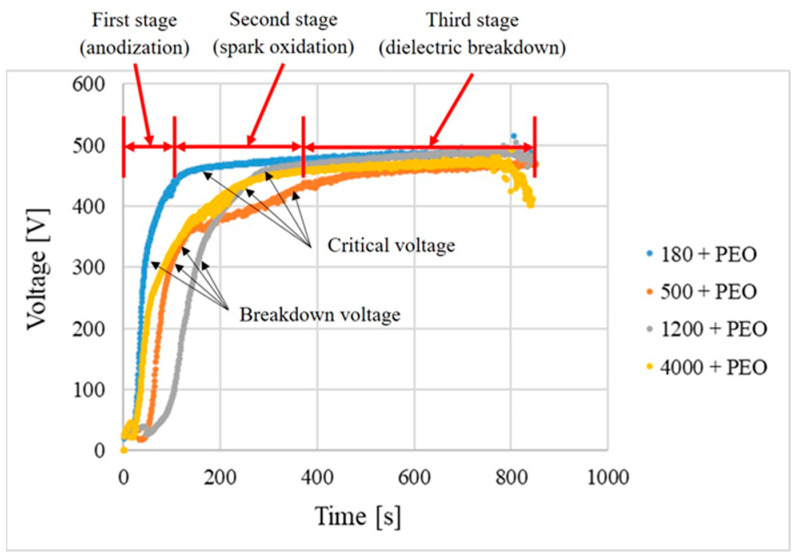
Evolution of voltage–time dependence.

In order to verify the effect of the mechanical pretreatment of the Mg alloy AZ80 before the PEO process itself, and then to determine its influence on the quality, formation, and exposure time of the samples in the PEO electrolyte, the time–voltage dependence was recorded for each type of grinding. Figure 5 shows the time–voltage dependence for all four types of mechanical pretreatments, which shows that the shape of all four curves is similar. During the PEO process, the increasing voltage was associated with the sparking phenomenon. The values of peak voltage for all samples were 470 ± 20 V in the last stage. With the presence of breakdown and critical voltage more or less similar for all the recordings, the obtained curves can be divided into three stages. In the first stage (anodization), it can be seen that the sharp increase in voltage is accompanied by an increase in time. The rate of the initial voltage rise indicates a rapid passivation of the sample surface at the beginning of the oxidation process. After 2 min, the voltage increase slows down. This fact was also observed for a submerged material AZ80 (Figure 5). However, the stabilization of the voltage rate after two minutes was observed only for samples 180 + PEO and 500 + PEO. On the other hand, for samples 1200 + PEO and 4000 + PEO, the voltage increase stabilized after 200 *s*. Based on these findings, this could be attributed to the influence of surface roughness on the voltage evolution over time. Another typical feature of this stage is an intense gas release with a large number of tiny sparks appearing and moving rapidly over the surface of the sample. This stage is typical of the formation of a thin, transparent, passive layer. In addition, oxygen evolution reactions partially took place at the anode surface just before the dielectric breakdown of the protective layer and after the plasma discharge [30]. The oxygen molecules are formed by the oxidation of water and adsorbed uniformly on the metal surface together with hydroxyl anions. The movement of the anions and cations is ensured by the electric field and the coating thickness is increased. For the following second stage (spark oxidation) of the PEO process, the continuous increase in the voltage is typical. However, the slope of the curves (Figure 5) is less steep compared to the anodization stage. It is described as a transition between the breakdown voltage and the critical voltage, where an enormous number of tiny white discharges occur on the entire anode surface. After the critical voltage, the PEO process enters the third stage (dielectric breakdown), which lasts until the end of the entire PEO treatment. The onset of this stage itself is essentially characterized by a flattening of the voltage–time curves with a gradual transformation of the discharges not only in colour appearance, from white to dazzling bright yellow, but also the increase in size and acoustic emission was evident [31,32].

### 3.4. SEM and EDS Analysis of Magnesium Alloy AZ80 with PEO Coating

**Figure 6:**
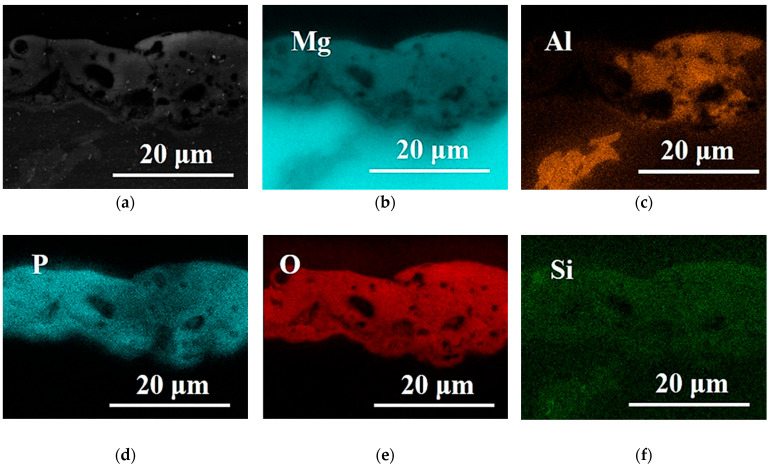
EDS analysis—(**a**) EDX mapping and distribution of (**b**) Mg, (**c**) Al, (**d**) P, (**e**) O and (**f**) Si of PEO coating created on AZ80 alloy in the electrolyte containing 12 g/L of Na_3_PO_4_·12H_2_O.

Figure 6a–f shows the EDS analysis of the elements present in the cross-section of AZ80 alloy with PEO coating. From the XRD analysis performed in other articles [12,32], it was found that the PEO coating formed in the phosphate electrolyte consists of Mg_3_(PO_4_)_2_ and MgO. This fact was confirmed by the EDS analysis shown in Figure 6b,d,e where the main elements of the PEO coating were phosphorus, oxygen, and magnesium. Moreover, the amount of phosphorus and oxygen is evenly distributed in the PEO coating.

**Figure 7:**
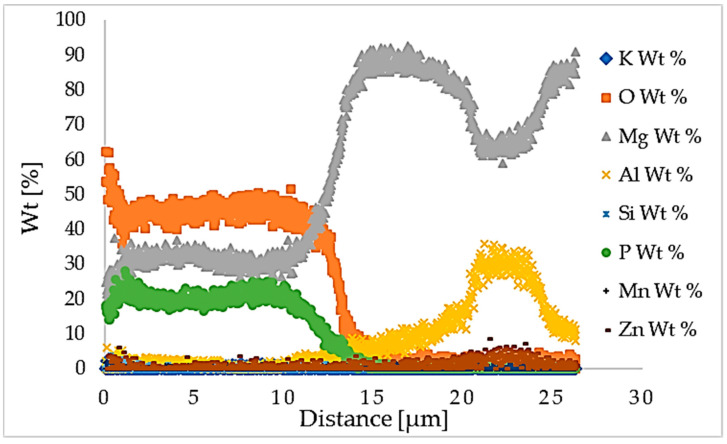
EDS line scan.

Figure 7 shows the graph of EDS line scan, which represents the total percentage content of elements in the AZ80 Mg alloy with PEO coating. The chemical composition of the PEO coating, with the main elements (oxygen, magnesium, and phosphorus), was confirmed by the weight percentage in the line scan graph.

The porous structure is a typical feature for PEO coatings. Basically, the amount and size of the pores are influenced by various aspects, such as the chemical composition of the coated material, the preparation time, and the concentration of certain elements added to the PEO bath. However, the largest pores are typical for sites consisting of intermetallic phases, which are associated with a higher occurrence of discharges. On the other hand, the presence of molten oxides in the cold electrolyte and the gas bubbles generated during discharges cause the formation of micropores and microcracks [4,17].

The average thickness and roughness parameters of PEO coatings are shown in Table 2. From the average thickness values, it can be seen that sample 1200—PEO has the highest average thickness value. In contrast, sample 4000—PEO has the lowest average thickness value. From the results, it is clear that as the roughness of the base material increased, the thickness of the coating also increased. However, the PEO coating formed on the metal surface is not uniform. Therefore, it is difficult to say exactly whether the roughness parameters R_a_ and R_z_ have a significant effect on the coating thickness [15]. In addition, the roughness parameters R_a_ and R_z_ gradually decreased with increasing grain size. This fact was clear even before the actual measurement.

**Figure 8:**
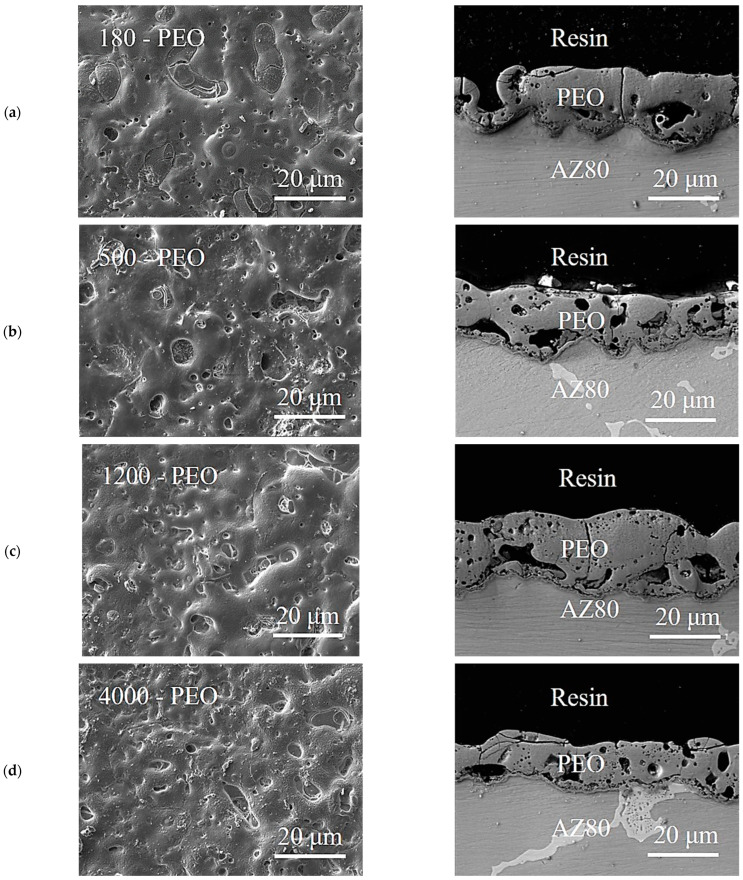
Surface morphology and cross-section images of the PEO coating prepared on variously mechanically pretreated samples—(**a**) 180—PEO, (**b**) 500—PEO, (**c**) 1200—PEO, and (**d**) 4000—PEO.

In Figure 8a–d the surface morphologies and cross-sections of the PEO coatings with different roughnesses of the base material are shown. In both the surface morphologies and the cross-sectional images, pores and microcracks with different dimensions and shapes were observed in all samples. However, it is difficult to say whether the size of these drawbacks increases simultaneously with the surface roughness of AZ80. On the other hand, their formation is a typical feature of the PEO process and is caused by oxygen production, which may be associated with crystallization of amorphous elements in the inner film. In the case of magnesium alloys, it is unlikely that high porosity of PEO coatings can be prevented. The electrical parameters play an important role in PEO formation, so it is very difficult to change them without changing the behavior of the PEO coating [33]. The pore size is affected by many factors, such as the applied current density, the duration of the PEO bath itself, and the applied voltage also plays an important role [34]. During the growth of PEO coating on Mg alloys, the main electrochemical reactions (2)–(5), which take place at the interface of the coating using a phosphate electrolyte, form protective compounds Mg(OH_2_), MgO, and ions in the form of Mg and ${PO}_{4}^{3-}$ [35].

(2) $$Mg\to {Mg}^{2+}+{2e}^{-},$$

(3) $${Mg}^{2+}+{2OH}^{-}\to {Mg\left(OH\right)}_{2},$$

(4) $${Mg\left(OH\right)}_{2}\to MgO+{2H}_{2}O,$$

(5) $${3Mg}^{2+}+{2PO}_{4}^{3-}\to {Mg}_{3}{(PO}_{4}{)}_{2}.$$

**Figure 9:**
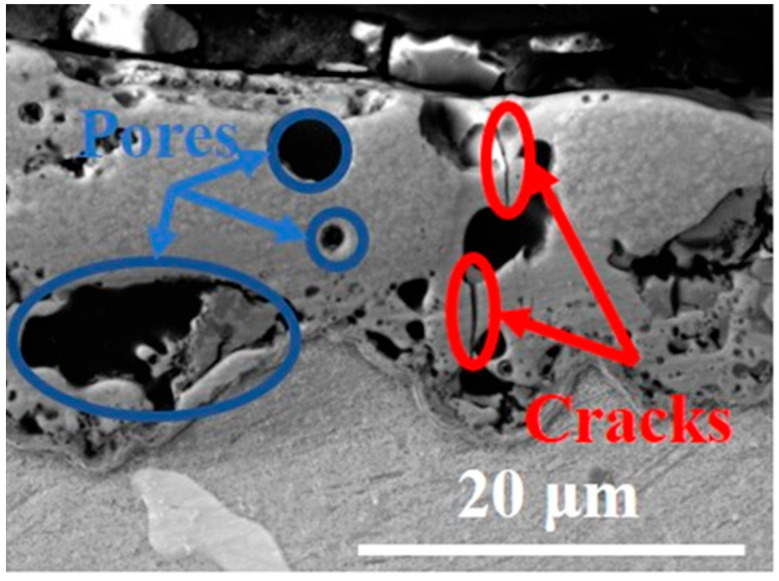
SEM image of pores and cracks on the PEO coating.

One of the main objectives of this research was to determine the effect of mechanical pretreatment on surface roughness and thickness of PEO coatings applied on AZ80 magnesium alloy. According to the results obtained by the SEM images (Figure 8a–d), it is obvious that the porous structure with a huge amount of pores, cracks, and other defects was present in the case of all samples that were treated with PEO coatings (Figure 9). The appearance of these defects on the PEO coating itself is a common feature of the PEO process. This is due to the fact that the entire formation of the PEO coating is accompanied by various chemical reactions that are the direct result of the voltage, the applied constant current, and an increasing temperature of the PEO electrolyte. The thickness of the PEO coating also has a great influence on the corrosion behavior of the magnesium alloy. However, the formation of an adhesive oxide ceramic coating is the best way to produce adequate surface protection in the case of magnesium alloys. This finding is basically supported by many studies [36,37].

In our case, the PEO coating itself consists of phophate compounds, especially Mg_3_(PO_4_)_2_ and PO_4_^−3^, which tend to deposit on the oxide surface. Thanks to these chemical particles, the corrosion behavior of the magnesium alloy is improved. Moreover, the combinations of compounds such as Mg(OH)_2_ and MgO are also involved in the formation of the protective film [38].

### 3.5. Corrosion Testing—Electrochemical Impedance Spectroscopy of Variously Pretreated Samples and Samples with PEO Coatings

**Figure 10:**
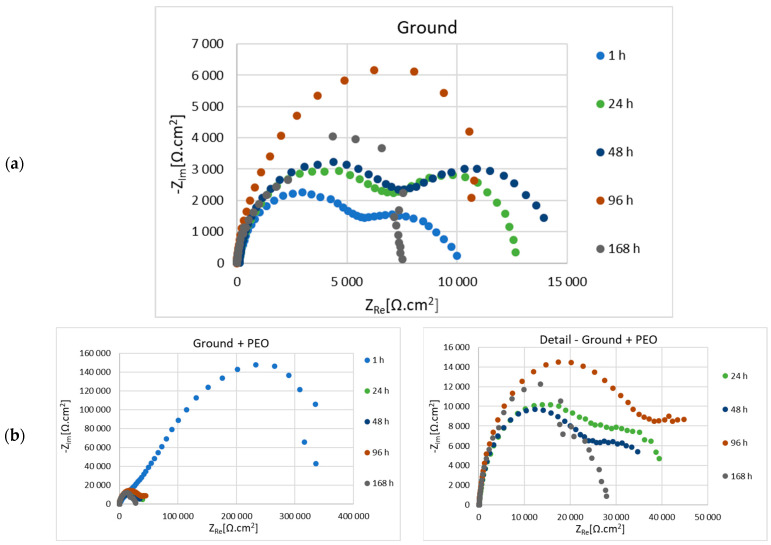
Nyquist diagrams of 180—ground (**a**) and 180—ground + PEO (**b**) AZ80 Mg alloy measured in 0.1 M NaCl.

**Figure 11:**
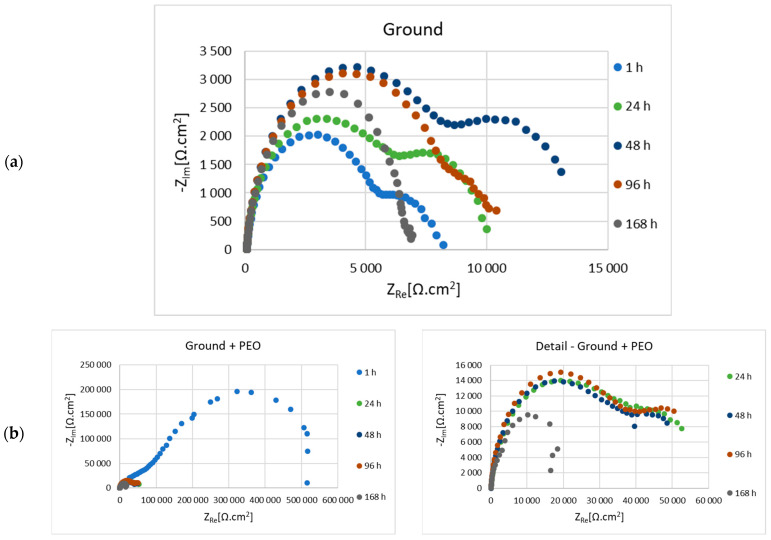
Nyquist diagrams of 500—ground (**a**) and 500—ground + PEO (**b**) AZ80 Mg alloy measured in 0.1 M NaCl.

**Figure 12:**
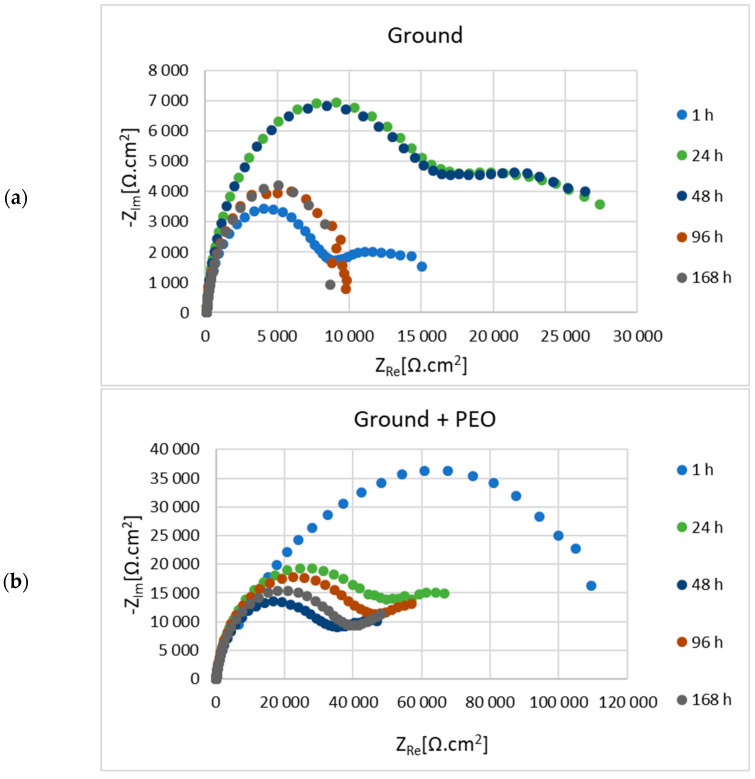
Nyquist diagrams of 1200—ground (**a**) and 1200—ground + PEO (**b**) AZ80 Mg alloy measured in 0.1 M NaCl.

**Figure 13:**
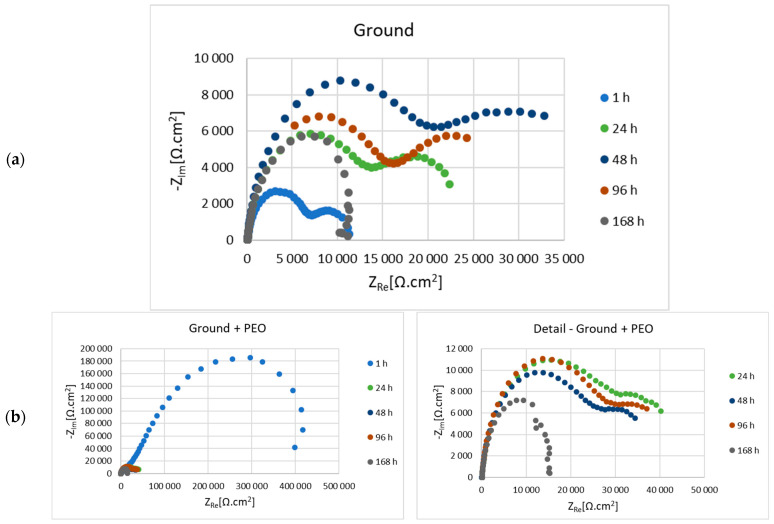
Nyquist diagrams of 4000—ground (**a**) and 4000—ground + PEO (**b**) AZ80 Mg alloy measured in 0.1 M NaCl.

Before the actual PEO process, the as-cast samples of Mg alloy AZ80 with ground surfaces—p180, p500, p1200 and p4000, respectively—were subjected to electrochemical impedance spectroscopy (EIS) measurements in 0.1 M NaCl solution. The results of electrochemical corrosion characteristics for ground and ground + PEO surfaces are shown in Figure 10a,b, Figure 11a,b, Figure 12a,b and Figure 13a,b. Analysis of these values was performed using Nyquist plots by a simple Randels circuit with one capacitive loop and a more complex circuit with two capacitive loops.

Nyquist diagrams for 180—ground and 180—ground + PEO AZ80 are shown in Figure 10a,b, and the corresponding values of electrochemical properties are given in Table 3 and Table 4. From the comparison of the ground and coated surfaces, the highest value of polarization resistance (Rp) (314,735 Ω.cm^2^) was found after 1 h exposure for the surface with PEO coating. Despite this fact, in the following phases, both types of surfaces are represented by increasing and decreasing resistance in a zig-zag shape. Nevertheless, in the last measurement, a higher value of Rp (36,526 Ω.cm^2^) was obtained from the coated surface.

Figure 11a,b shows the Nyquist plots for the 500—ground and 500—ground + PEO samples, and the values of the electrochemical corrosion characteristics are shown in Table 5 and Table 6, respectively. The higher value of Rp (463,127 Ω.cm^2^) after the initial phase is observed for the sample with PEO coating. The trend of increasing and decreasing resistivity values is also observed in this case. The resulting Rp value was higher for the coated sample (13,376 Ω.cm^2^), almost three times higher than the uncoated sample (5483 Ω.cm^2^).

In Figure 12a,b is shown Nyquist plots for the 1200—ground and 1200—ground + PEO samples, while the values of the evaluated electrochemical properties are listed in Table 7 and Table 8. The value of Rp (127,095 Ω.cm^2^) is 9 times higher in the case of sample 1200—ground + PEO sample than in the case of 1200—ground (13,537 Ω.cm^2^) after 1 h of exposure to 0.1 M NaCl environment. The Rp value is characterized by an increasing and decreasing trend for the uncoated and coated samples, as was observed for the previous samples. However, the final Rp value of the sample 1200—ground + PEO is 52,580 Ω.cm^2^, which is 7 times higher than sample 1200—ground.

Figure 13a,b shows the Nyquist plots for the 4000—ground and 4000—ground + PEO samples. Table 9 and Table 10 listed the values of the electrochemical corrosion properties. The highest value of Rp = 501,464 Ω.cm^2^ was obtained in the initial stage of exposure from sample 4000—ground + PEO. The trend with increasing and decreasing Rp value remained the same. After the last exposure phase, a higher Rp value (13,103 Ω.cm^2^) was obtained from sample 4000—ground + PEO.

Nowadays the most commonly used magnesium alloys are those alloyed with aluminum, zinc, and manganese. The content of impurities such as Fe, Cu, and Si should not exceed the percentage range of 0.4—0.6% [39]. The element aluminum plays a very important role in the corrosion resistance of magnesium alloys. This is due to the fact that aluminum forms the intermetallic phase β (Mg_17_Al_12_) with magnesium. The corrosion resistance of this phase in magnesium alloy is better compared to α-phase. The other type of particles of the second phase, which also has a great influence on the corrosion behavior, is the Mn-Al type. Based on this fact, it is obvious that the corrosion behavior of as-cast alloys is affected by second-phase particles precipitated during casting. Therefore, it is clear that the aluminum content in the chemical composition of the alloy plays an important role in corrosion resistance. Neverheless, the composition of the alloy and the cooling rate during the casting process are the main influences that directly affect the volume fraction and morphology of these particles [40]. These second phase particles were found to affect not only the corrosion behavior of magnesium alloys, but also their mechanical properties [41,42,43]. Both Mg_17_Al_12_ and Mn-Al particles behave cathodically over a range of pH values, leading to the formation of galvanic couples and subsequent formation of pits. Pardo et al. suggested that corrosion begins at the matrix interface and propagates through the nucleation and growth of the protective Mg(OH)_2_ layer in the next step [44].

In our case, magnesium alloy AZ80 was used as the experimental material. The effect of the different emery papers on the corrosion resistance of the ground and coated specimens was evaluated in an aggressive (chloride-containing) environment consisting of 0.1 M NaCl. To further assess the effect of the granin size of the emery papers on the corrosion behavior, electrochemical impedance spectroscopy (EIS) was performed for the ground and coated surfaces. From the R_p_ values shown in Table 3, Table 5, Table 7 and Table 9, it can be seen that the formation and growth of the corrosion products (MgO, Mg(OH)_2_, and MgCl_2_) gradually peaked at R_p_ with increasing exposure time. After 48 h of exposure time to an aggressive cloride environment, the peak values of R_p_ were reached for samples 180—ground (14,805 Ω.cm^2^), 500—ground (14,258 Ω.cm^2^), and 4000—ground (31,633 Ω.cm^2^). For sample 1200—ground, the peak value of R_p_ (28,927 Ω.cm^2^) was resched after 48 h exposure time. The polarization resistance of samples 180—ground, 500—ground, 1200—ground, and 4000—ground after 168 h exposure time had values of 5730 Ω.cm^2^, 5483 Ω.cm^2^, 7190 Ω.cm^2^, and 8713 Ω.cm^2^, respectively. This fact can be attributed to the effect of finer surface texture [15]. Song et al. found that the protective films formed on the surface of magnesium alloys originate from the alloying elements (Al, Mn, or Zn) with which Mg is alloyed. When the magnesium alloy surface is exposed to the atmosphere, these conditions lead to the formation of secondary phases, such as the Mg-Al phase. The occurrence of these phases supports the formation of a stable passive film. Therefore, the corrosion behavior of the film is influenced by many aspects, such as the chemical composition of the electrolyte and the base material, the exposure time, or various defects [39]. In contrast to the ground surfaces, the polarization resistance for all coated surfaces had values of 314,735 Ω.cm^2^, 463,127 Ω.cm^2^, 127,095 Ω.cm^2^, and 501,464 Ω.cm^2^, respectively, after 1 h of exposure time in 0.1 M NaCl solution (180—ground + PEO, 500—ground + PEO, 1200—ground + PEO, and 4000—ground + PEO) (Table 4, Table 6, Table 8 and Table 10). Moreover, the R_p_ values were significantly higher for all coated surfaces compared to the ground surfaces. This fact was also confirmed in the research work of Štrbák et al. [17]. Although the R_p_ values decreased with increasing exposure time for all samples, the R_p_ values of the coated surfaces remained significantly higher than those of the ground surfaces. Throughout the exposure time of the sample in an aggressive environment, the presence of Cl^−^ ions caused the gradual penetration of the protective Mg_3_(PO_4_)_2_ layer, formed on the base material. However, the values of R_p_ (Table 3, Table 4, Table 5, Table 6, Table 7, Table 8, Table 9 and Table 10) are characterized by a zig-zag behavior in all samples. This type of behavior is caused by the gradual penetration of the aggressive particles of the cloride environment onto the surface of the coated material with the simultaneous formation of passive films (Mg(OH)_2_ and MgCl_2_). During the exposure period, the size and mass of these defects gradually increase and they have a tendency to form larger voids, which leads to a decrease in the R_p_ value. Nonetheless, after a certain exposure time, these voids are partially closed by the passive films, leading to an increase in the R_p_ value [45]. After 168 h of exposure time (Table 4, Table 6, Table 8 and Table 10) the polarization resistance of the samples 180—ground + PEO, 500—ground + PEO, 1200—ground + PEO, and 4000—ground + PEO decreased and had values of 36,526 Ω.cm^2^, 13,376 Ω.cm^2^, 52,580 Ω.cm^2^, and 13,103 Ω.cm^2^, respectively. The highest R_p_ value was obtained after 168 h of exposure time for sample 1200—ground + PEO. This could be due to the influence of the condition of the base material on which the PEO coating was formed. On the other hand, the R_p_ value was significantly lower in the case of sample 4000—ground + PEO. Therefore, a surface condition with insufficient heterogeneity is not suitable for coating.

The usual composition of PEO coatings consists of an outer and an inner layer, expressed by the corresponding partial resistances R_1_ and R_2_. The function of the inner layer is to form an obstacle for the aggressive media and to increase the corrosion resistance [12]. Based on the results, it is clear that the protection of this layer decreased significantly during the exposure time for all samples. The gradual penetration of the electrolyte through the coating was caused by its lower compactness, which led to chemical dissolution, and consequently, to a decrease in resistance after only 1 h of exposure. For this reason, in the period from 2 to 168 h, R_1_ corresponds to the combined response of the outer and inner layers, while R_2_ is referred to as the charge transfer resistance [46]. However, the comparison of corrosion resistance by the inner layer showed that the values of ground + PEO are higher compared to ground samples.

The component n ranges from 0.6 to 1 in Table 3, Table 4, Table 5, Table 6, Table 7, Table 8, Table 9 and Table 10 and is attributed to the value of capacitor. Therefore, the element CPE in the equivalent circuit should be replaced by a capacitor with *n* = 1 [47]. The CPE_1_ and CPE_2_ values of ground and ground + PEO samples increase after each exposure time (Table 3, Table 4, Table 5, Table 6, Table 7, Table 8, Table 9 and Table 10), mainly for CPE_2_. It can be seen that after 168 h of exposure, the value of CPE_2_ increased for the samples 180—ground, 500—ground, 500—ground + PEO, 1200—ground + PEO, and 4000—ground + PEO. This fact was caused by the still ongoing corrosion reactions and enlargement of the active area. Nevertheless, the CPE_1_ and CPE_2_ values for the other ground and coated samples decreased slightly after 168 h of exposure.

### 3.6. Evaluation of Potentiodynamic Curves

**Figure 14:**
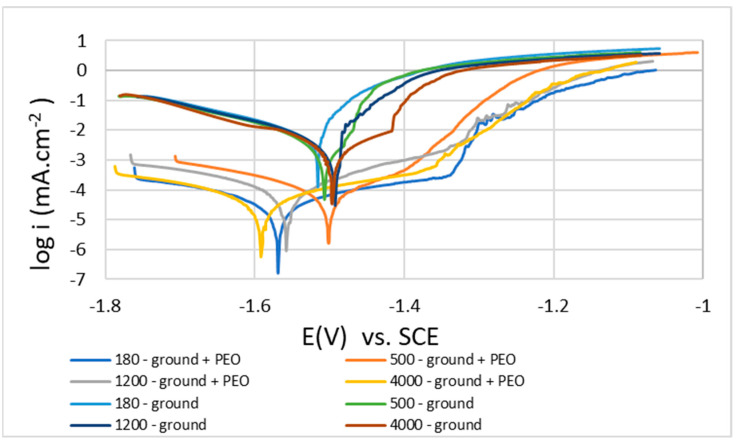
PD curves for uncoated and coated AZ80 Mg alloy surfaces measured in 0.1 M NaCl.

Figure 14 shows potentiodynamic polarization curves of uncoated and coated samples of Mg alloy AZ80 measured in 0.1 M NaCl solution. The analysis of these curves was performed by the Tafel extrapolation method, and then the curves were plotted on the semi-logarithmic scale. The PDP measurements were repeated three times for each type of surface pretreatment. From these measurements, the values of the following corrosion characteristics were determined: corrosion potential (E_corr_), corrosion current density (icorr), corrosion rate (r_corr_), and the inclinations of Tafel constants (β_a_ and β_c_), which are shown in Table 11 and Table 12. The thermodynamics of the corrosion reaction are described by E_corr_, while the kinetic side is described by i_corr_ and provides information on the rate of corrosion reactions [17].

Based on the results of the PDP tests (Table 11 and Table 12), it can be said that after comparing the ground surfaces with the ground surfaces + PEO coatings, it is obvious that the application of PEO coatings on all surfaces shifted the Ecorr values towards more negative values. This fact is typical for materials that have rougher oxide layers on their surfaces [15]. The most negative value of Ecorr (−1594 mV) was obtained by the sample 180—ground + PEO. However, for the corrosion current densities (i_corr_), the values obtained were significantly lower for all PEO surfaces compared to the ground surfaces. For all samples with PEO coatings, the determined decrease in icorr was two orders of magnitude lower. From the reached results, it can be seen that the most significant decrease in icorr occurred in the sample 180—ground + PEO, namely from 7.07 μA.cm^−2^ (180—ground) to 0.06 μA.cm^−2^. However, the other changes in the obtained results, namely from 5.12 μA.cm^−2^ to 0.08 μA.cm^−2^ for 500—ground + PEO, from 4.28 μA.cm^−2^ to 0.08 μA.cm^−2^ for 1200—ground + PEO, and from 3.56 μA.cm^−2^ to 0.06 μA.cm^−2^ 4000—ground + PEO, were also impressive. From the values of the corrosion rates (r_corr_), it can be seen (Table 11 and Table 12) that the r_corr_ is significantly lower in the case of the samples ground + PEO compared to the ground samples. It is obvious that the most significant decrease in r_corr_ value by almost 163 times was measured in the case of sample 180—ground + PEO, namely from 162 μmpy (180—ground) to 1.28 μmpy. The order of the other results obtained is as follows: from 195 μmpy to 1.86 μmpy for 1200—ground + PEO, from 117 μmpy to 1.83 for 500—ground + PEO, and from 81 μmpy to 1.41 μmpy for 400—ground + PEO.

## 4. Conclusions

In this study, the corrosion behavior and morphology of PEO-coated Mg alloy AZ80 were investigated, and the following conclusions can be drawn:

⮚ The PEO coating prepared by the PEO process on the as-cast surface of AZ80 +magnesium alloy exhibits typical pores and cracks of different sizes.
⮚ The application of PEO coatings on the samples mechanically pretreated with different emery papers showed a significant reduction (two orders of magnitude) in the value i_corr_ for all coated samples (from 7.07 μA.cm^−2^ to 0.06 μA.cm^−2^ for 180—ground + PEO, from 5.12 μA.cm^−2^ to 0.08 μA.cm^−2^ for 500—ground + PEO, from 5.12 μA.cm^−2^ to 0.08 μA.cm^−2^ for 500—ground + PEO, from 4.28 μA.cm^−2^ to 0.08 μA.cm^−2^ for 1200—ground + PEO, and from 3.56 μA.cm^−2^ to 0.06 μA.cm^−2^ 4000—ground + PEO). However, the most significant decrease almost of 118 times was recorded for sample 180—ground + PEO.
⮚ From a thermodynamic point of view, the value of E_corr_ shifted to a more negative value for all coated surfaces compared to the ground surfaces. The most negative value of Ecorr (-1594 mV) was obtained for sample 180—ground + PEO.
⮚ The higher corrosion resistance in terms of EIS measurements was achieved by all coated surfaces compared to the ground surfaces.
⮚ The R_p_ values are contrariwise proportional to icorr (a higher R_p_ value means a lower icorr value), therefore, the R_p_ differences in coated surfaces are due to the occurrence of heterogeneities and different dimensions of the samples.
⮚ The most significant increase in Rp of almost 61 times (from 7623 Ω.cm^2^ to 463,127 Ω.cm^2^) after 1 h of exposure in 0.1 M NaCl solution was observed for sample 500—ground + PEO.
⮚ After 168 h of exposure in 0.1 M NaCl solution, the highest R_p_ value (525,80 Ω.cm^2^) was obtained for sample 1200—ground + PEO.

## Figures and Tables

**Table 1:** Chemical composition of AZ80 magnesium alloy.

Element	Mg [%]	Al [%]	Zn [%]	Mn [%]	Cu [ppm]
AZ80	bal.	10.42	0.37	0.15	2.20

**Table 2:** Thickness and roughness parameters of mechanically pretreated samples.

Samples	Thickness [μm]	Roughness [μm] *R_a_ R_z_ R_sk_*		
		*R_a_*	*R_z_*	*R_sk_*
180—ground		1.19	10.55	−0.66
180—PEO	15.47 ± 2.51	1.05	3.73	0.37
500—ground		1.21	12.29	−0.21
500—PEO	15.01 ± 3.26	1.13	4.76	0.01
1200—ground		0.64	8.36	−0.27
1200—PEO	18.81 ± 6.13	0.67	3.05	−0.43
4000—ground		0.42	5.77	0.02
4000—PEO	14.06 ± 2.24	0.56	2.69	0.14

**Table 3:** Corrosion electrochemical characteristics of 180—ground AZ80 Mg alloy measured in 0.1 M NaCl.

Time	R_s_ (Ω·cm^2^)	R_1_ (Ω·cm^2^)	R_2_ (Ω·cm^2^)	R_p_ (Ω·cm^2^)	CPE_1_(F·s^n−1^·10^−6^)	CPE_2_ (F·s^n−1^·10^−6^)	n_1_	n_2_
1 h	83 ± 4	4643 ± 142	5657 ± 482	10,300 ± 624	23.5 ± 4.1	2.3 ± 0.5	0.9	1
24 h	81 ± 5	9974 ± 1172	1326 ± 267	11,300 ± 1439	8.3 ± 1.6	1.5 ± 0.2	1	0.7
48 h	85 ± 6	2743 ± 212	12,062 ± 2061	14,805 ± 2273	30.2 ± 7.1	8.9 ± 3.5	0.8	0.9
96 h	77 ± 8	7989 ± 1432		7989 ± 1432	13.9 ± 4.8		1	
168 h	79 ± 9	5730 ± 603		5730 ± 603	23.2 ± 6.3		1	

**Table 4:** Corrosion electrochemical characteristics of 180—ground + PEO AZ80 Mg alloy measured in 0.1 M NaCl.

Time	R_s_ (Ω·cm^2^)	R_1_ (Ω·cm^2^)	R_2_ (Ω·cm^2^)	R_p_ (Ω·cm^2^)	CPE_1_(F·s^n−1^·10^−6^)	CPE_2_ (F·s^n−1^·10^−6^)	n_1_	n_2_
1 h	125 ± 2	67,187 ± 15,982	247,548 ± 4100	314,735 ± 20,082	2.8 ± 0.9	7.6 ± 2.1	0.6	0.8
24 h	120 ± 7	43,653 ± 1111		43,653 ± 1111	73.5 ± 43.1		0.9	
48 h	112 ± 10	37,680 ± 1755		37,680 ± 1755	10.3 ± 0.8		0.9	
96 h	115 ± 8	45,412 ± 1205		45,412 ± 1205	26.7 ± 5.3		0.9	
168 h	107 ± 5	36,526 ± 8337		36,526 ± 8337	7.1 ± 1.6		0.9	

**Table 5:** Corrosion electrochemical characteristics of 500—ground AZ80 Mg alloy measured in 0.1 M NaCl.

Time	R_s_ (Ω·cm^2^)	R_1_ (Ω·cm^2^)	R_2_ (Ω·cm^2^)	R_p_ (Ω·cm^2^)	CPE_1_(F·s^n−1^·10^−6^)	CPE_2_ (F·s^n−1^·10^−6^)	n_1_	n_2_
1 h	95 ± 1	4442 ± 730	3181 ± 15	7623 ± 745	15.1 ± 0.6	1.2 ± 0.7	0.9	0.7
24 h	92 ± 2	3166 ± 156	5932 ± 125	9098 ± 281	15.2 ± 5.2	28.4 ± 6.1	0.9	0.9
48 h	93 ± 4	6064 ± 1106	8194 ± 353	14,258 ± 1459	21.2 ± 8.9	32.5 ± 14.9	0.8	0.8
96 h	94 ± 2	7955 ± 1002		7955 ± 1002	27.1 ± 6.8		0.8	
168 h	93 ± 2	5486 ± 36		5483 ± 36	31.8 ± 6.1		0.8	

**Table 6:** Corrosion electrochemical characteristics of 500—ground + PEO AZ80 Mg alloy measured in 0.1 M NaCl.

Time	R_s_ (Ω·cm^2^)	R_1_ (Ω·cm^2^)	R_2_ (Ω·cm^2^)	R_p_ (Ω·cm^2^)	CPE_1_(F·s^n−1^·10^−6^)	CPE_2_ (F·s^n−1^·10^−6^)	n_1_	n_2_
1 h	125 ± 5	459,645 ± 31,747	3482 ± 169	463,127 ± 31,916	1.9 ± 0.8	25.9 ± 4.7	0.6	0.7
24 h	120 ± 7	55,807 ± 763	13,081 ± 423	68,888 ± 1186	4.7 ± 1.3	28.4 ± 2.6	0.9	0.9
48 h	123 ± 4	41,651 ± 588	7512 ± 1844	49,163 ± 2432	9.9 ± 2.8	2.4 ± 1.6	0.8	0.7
96 h	122 ± 5	30,664 ± 585	25,603 ± 1814	56,267 ± 2399	14.4 ± 3.5	2.8 ± 1.4	0.9	0.8
168 h	123 ± 6	13,376 ± 1912		13,376 ± 1912	2.9 ± 1.9		1	

**Table 7:** Corrosion electrochemical characteristics of 1200—ground AZ80 Mg alloy measured in 0.1 M NaCl.

Time	R_s_ (Ω·cm^2^)	R_1_ (Ω·cm^2^)	R_2_ (Ω·cm^2^)	R_p_ (Ω·cm^2^)	CPE_1_(F·s^n−1^·10^−6^)	CPE_2_ (F·s^n−1^·10^−6^)	n_1_	n_2_
1 h	93 ± 3	8429 ± 1324	5108 ± 182	13,537 ± 1506	10.4 ± 2.9	44.2 ± 7.7	1	1
24 h	95 ± 1	7429 ± 147	21,498 ± 1346	28,927 ± 1493	12.3 ± 0.6	6.8 ± 1.2	0.9	0.8
48 h	94 ± 2	12,118 ± 166	11,036 ± 165	23,154 ± 331	35.2 ± 1.3	4.6 ± 0.9	0.9	0.8
96 h	93 ± 1	11,589 ± 1353		11,589 ± 1353	63.2 ± 2.8		0.8	
168 h	92 ± 3	7190 ± 759		7190 ± 759	45.2 ± 16.1		0.8	

**Table 8:** Corrosion electrochemical characteristics of 1200—ground + PEO AZ80 Mg alloy measured in 0.1 M NaCl.

Time	R_s_ (Ω·cm^2^)	R_1_ (Ω·cm^2^)	R_2_ (Ω·cm^2^)	R_p_ (Ω·cm^2^)	CPE_1_(F·s^n−1^·10^−6^)	CPE_2_ (F·s^n−1^·10^−6^)	n_1_	n_2_
1 h	115 ± 3	10,3739 ± 4931	23,356 ± 1462	127,095 ± 6393	6.4 ± 0.2	5.3 ± 0.3	0.6	0.7
24 h	105 ± 1	67,353 ± 360	3845 ± 1018	71,198 ± 1378	17.8 ± 11.8	0.6 ± 0.1	0.9	0.8
48 h	107 ± 2	13,274 ± 2862	41,600 ± 887	54,874 ± 3749	17.7 ± 8.7	0.2 ± 0.1	0.9	1
96 h	103 ± 3	66,708 ± 1383	5489 ± 858	72,197 ± 2241	16.5 ± 1.8	0.3 ± 0.1	0.9	1
168 h	101 ± 5	34,878 ± 3717	20,638 ± 16	52,580 ± 2705	4.3 ± 0.8	26.8 ± 2.1	1	0.8

**Table 9:** Corrosion electrochemical characteristics of 4000—ground AZ80 Mg alloy measured in 0.1 M NaCl.

Time	R_s_ (Ω·cm^2^)	R_1_ (Ω·cm^2^)	R_2_ (Ω·cm^2^)	R_p_ (Ω·cm^2^)	CPE_1_(F·s^n−1^·10^−6^)	CPE_2_ (F·s^n−1^·10^−6^)	n_1_	n_2_
1 h	99 ± 2	1229 ± 30	7492 ± 2017	8721 ± 2047	12.1 ± 4.1	0.3 ± 0.1	0.8	0.8
24 h	98 ± 1	2280 ± 153	23,205 ± 155	25,485 ± 308	10.2 ± 0.1	17.3 ± 9.8	0.9	0.8
48 h	98 ± 1	1175 ± 18	30,458 ± 5303	31,633 ± 5321	24.7 ± 7.1	4.9 ± 2.9	1	0.8
96 h	95 ± 4	7270 ± 588	17,858 ± 226	25,128 ± 814	19.7 ± 0.1	1.9 ± 1.2	0.9	0.7
168 h	92 ± 8	8713 ± 1349		8713 ± 1349	18.1 ± 3.2		0.7	

**Table 10:** Corrosion electrochemical characteristics of 4000—ground + PEO Mg alloy measured in 0.1 M NaCl.

Time	R_s_ (Ω·cm^2^)	R_1_ (Ω·cm^2^)	R_2_ (Ω·cm^2^)	R_p_ (Ω·cm^2^)	CPE_1_(F·s^n−1^·10^−6^)	CPE_2_ (F·s^n−1^·10^−6^)	n_1_	n_2_
1 h	120 ± 2	24,084 ± 3152	477,380 ± 22,041	501,464 ± 25,193	3.6 ± 1.3	0.2 ± 0.1	0.7	0.9
24 h	111 ± 1	39,744 ± 2343	6991 ± 411	46,735 ± 2754	7.4 ± 0.4	38.1 ± 3.4	0.9	1
48 h	112 ± 1	29,160 ± 1705	12,664 ± 2827	41,824 ± 4532	5.4 ± 0.1	0.5 ± 0.3	0.9	0.8
96 h	109 ± 1	4066 ± 1	29,165 ± 5066	33,231 ± 5067	15.5 ± 0.4	1.2 ± 0.1	0.9	0.6
168 h	105 ± 1	13,103 ± 2932		13,103 ± 2932	9.9 ± 1.6		0.9	

**Table 11:** Electrochemical characteristics of uncoated Mg alloy surfaces measured in 0.1 M NaCl.

Corrosion Characteristics	E_corr_ [mV vs. SCE]	i_corr_ [μA·cm^−2^]	β_c_ [mV/dec.]	β_a_ [mV/dec.]	r_corr_ [μmpy]
180—ground	−1503 ± 30	7.07 ± 0.66	169 ± 7	36 ± 2	162 ± 15
500—ground	−1514 ± 15	5.12 ± 2.45	157 ± 22	45 ± 7	117 ± 56
1200—ground	−1495 ± 13	4.28 ± 1.14	165 ± 14	54 ± 16	195 ± 52
4000—ground	−1498 ± 8	3.56 ± 0.74	169 ± 32	94 ± 18	81 ± 17

**Table 12:** Electrochemical characteristics of coated Mg alloy surfaces measured in 0.1 M NaCl.

Corrosion Characteristics	E_corr_ [mV vs. SCE]	i_corr_ [μA·cm^−2^]	β_c_ [mV/dec.]	β_a_ [mV/dec.]	r_corr_ [μmpy]
180—ground + PEO	−1594 ± 63	0.06 ± 0.02	202 ± 12	203 ± 31	1.28 ± 0.47
500—ground + PEO	−1533 ± 63	0.08 ± 0.01	177 ± 15	183 ± 28	1.83 ± 0.12
1200—ground + PEO	−1533 ± 22	0.08 ± 0.02	193 ± 2	162 ± 27	1.86 ± 0.34
4000—ground + PEO	−1571 ± 34	0.06 ± 0.03	203 ± 6	234 ± 31	1.41 ± 0.64

## Data Availability

Data will be made available on request.
